# Automated astatination of biomolecules – a stepping stone towards multicenter clinical trials

**DOI:** 10.1038/srep12025

**Published:** 2015-07-14

**Authors:** Emma Aneheim, Per Albertsson, Tom Bäck, Holger Jensen, Stig Palm, Sture Lindegren

**Affiliations:** 1 Targeted Alpha Therapy, Department of Radiation Physics, Sahlgrenska Academy at Gothenburg University, Gothenburg 41345, Sweden; 2 PET and Cyclotron unit, KF3982, Copenhagen University Hospital, Copenhagen, Denmark

## Abstract

To facilitate multicentre clinical studies on targeted alpha therapy, it is necessary to develop an automated, on-site procedure for conjugating rare, short-lived, alpha-emitting radionuclides to biomolecules. Astatine-211 is one of the few alpha-emitting nuclides with appropriate chemical and physical properties for use in targeted therapies for cancer. Due to the very short range of the emitted α-particles, this therapy is particularly suited to treating occult, disseminated cancers. Astatine is not intrinsically tumour-specific; therefore, it requires an appropriate tumour-specific targeting vector, which can guide the radiation to the cancer cells. Consequently, an appropriate method is required for coupling the nuclide to the vector. To increase the availability of astatine-211 radiopharmaceuticals for targeted alpha therapy, their production should be automated. Here, we present a method that combines dry distillation of astatine-211 and a synthesis module for producing radiopharmaceuticals into a process platform. This platform will standardize production of astatinated radiopharmaceuticals, and hence, it will facilitate large clinical studies focused on this promising, but chemically challenging, alpha-emitting radionuclide. In this work, we describe the process platform, and we demonstrate the production of both astaine-211, for preclinical use, and astatine-211 labelled antibodies.

Adjuvant systemic chemotherapy for occult micrometastatic residual disease can increase survival, to some extent, after locoregional therapy of breast, colorectal, and ovarian cancers. However, the majority of patients are not cured. Therefore, there is a need to develop new therapeutic approaches. Among several new approaches currently under investigation, radioimmunotherapy with alpha-particle-emitting radionuclides has emerged as a promising application^1^,^2^.

Targeted alpha therapy takes advantage of the short tissue range (50–100 μm) of alpha particles. Thus, when these nuclides are targeted to malignant cells, they deliver a high local, extremely cytotoxic, radiation dose to the tumour, while surrounding healthy tissue is spared. This feature facilitates the treatment of disseminated cancers, such as micro tumours or single malignant cells. Recently, this therapy has become truly targeted, due to the increasing number of available tumour-specific vectors.

Astatine-211 (At-211) is one of the few rare, alpha-emitting radionuclides with suitable physical properties (7.2 h half-life and 100% alpha emission in its total decay) for applications within targeted alpha therapy^2^,^3^. Several preclinical studies that investigated At-211 for treating micro metastasis have been conducted with the free halide (i.e., astatide) or with At-211-labelled proteins, such as antibodies or peptides^4^,^5^. Promising preclinical results were obtained with astatinated antibodies, and two clinical phase I studies have emerged from those results^6^,^7^.

Astatine-211 is produced artificially in suitable cyclotrons by irradiating stable bismuth with 28 MeV α-particles in the ^209^Bi(α,2n)^211^At reaction. After cyclotron production, At-211 must be converted to a chemically useful form. This can be achieved by isolating At-211 molecules, by wet extraction^8^ or by dry distillation^9^, from the irradiated target material. Then, the At-211 can be subjected to chemical coupling reactions, and can serve as a component in radiopharmaceuticals.

The main drawback of current methods for producing At-211 radiopharmaceuticals is that the chemistry includes a series of manual steps, where the final result depends on the hands-on skills of laboratory personnel. Furthermore, because astatine is a very rare element and its isotopes only exist as short-lived radionuclides (longest half-life 8.3 h), the chemical properties of astatine are largely unknown. Although the manual method of synthesizing At-211 radiopharmaceuticals may be efficient, future progress in preclinical research and, in particular, clinical advancements with At-211 rely on further improving and developing the radiochemistry. The most common method for synthesizing astatinated biomolecules comprises two steps; first, a reagent is radiolabelled; then, the radiolabelled reagent is conjugated to a biomolecule^10^. However, this strategy frequently produces low yields and poor final quality. It is recognized that these problems are caused by radiolytic effects in the reacting solvents^11^,^12^. To overcome this issue, a new method was developed for synthesizing At-211 labelled biomolecules^13^. This method is similar to chelate chemistry, because a labelling reagent and the biomolecule are conjugated to form a complex, in advance of the radiolabelling. In this method, there is only one radiochemical step involved in the synthesis; thus, the radiation dose absorbed by the conjugate is drastically reduced. This new synthetic method makes it possible to convert the whole production process from a manual, step-wise methodology into a fully automated procedure. In previous work, the synthesis process was modified and scaled up to allow its implementation in a synthesis module^14^. In the present work, we describe a fully automatic platform for producing astatinated biomolecules. Then, we demonstrate the production of At-211 in a chemically useful form for preclinical work, and we demonstrate the synthesis of At-211-labelled antibodies.

## Results

**Figure 1:**
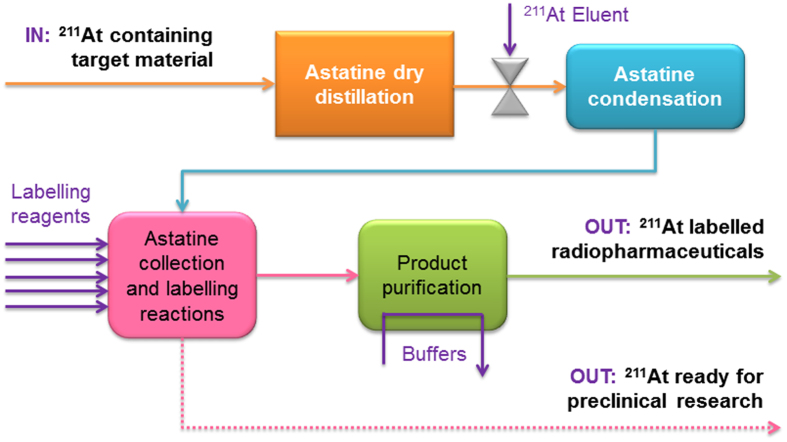
Schematic of the radiosynthesis process platform for producing ^211^At and ^211^At-labelled radiopharmaceuticals.

Figure 1 illustrates the components of a platform for the automated radiosynthesis of astatinated proteins and peptides. In this platform, At-211 is converted from a solid form, embedded in the irradiated bismuth target material, into a chemically useful (free) form.

**Figure 2:**
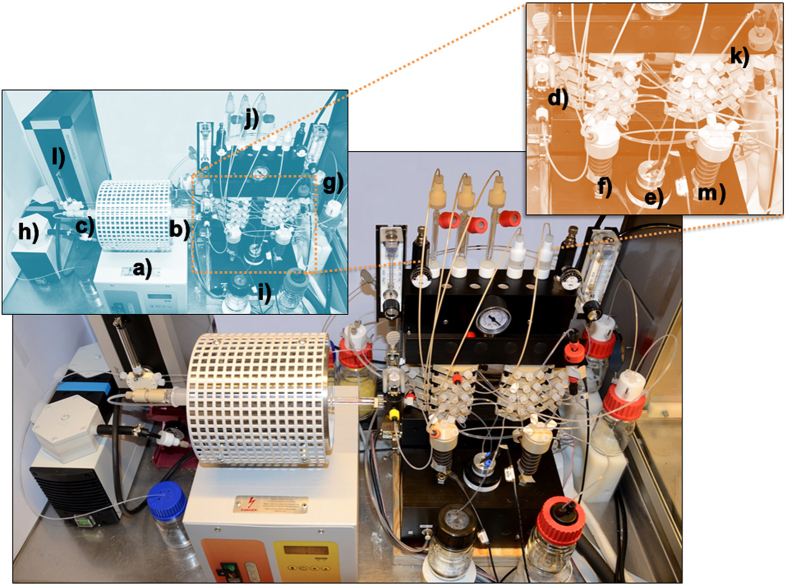
The automated process platform is set up inside a glovebox. The platform components are labelled in the upper boxes. The platform consists of (**a**) a tube furnace for At-211 dry distillation at 700 °C; (**b**) a quartz glass oven; the oven is fitted with a flow-through quartz glass stopper (**c,d**) three-way valve for capillary elution; (**e**) a cryotrap for astatine capture; (**f**) a reaction vial for radiolabelling compounds with At-211; (**g**) astatine capture traps (expansion trap, a sulphite scrub container, and another expansion trap); (**h**) a vacuum pump with a capacity of 10 l/min; (**i,j**) buffer and reagent storage containers; (**k**) an on-line purification column (size exclusion) with an attached silicon PIN diode detector; (**l**) a syringe dispenser, and (**m**) a product vial.

The automated platform (Fig. 2) comprises a tube furnace with quartz glassware for distillation, which is connected in tandem to a radiopharmaceutical synthesis module. Both the distillation procedure and the synthesis module are remotely controlled by a single computer software program. To increase radiation safety, the system was adapted to fit inside a glovebox or a small lead-shielded, hot-cell to minimize exposure to the volatile, radioactive astatine. This compact platform is very versatile as it can accommodate any type of target used for the cyclotron production of astatine. This, since the target material is separated from the target backing before being placed in the furnace. This target material separation step can be incorporated into the automated process, but in any case, it should be performed inside the glovebox or hot-cell to maintain radiation safety. Throughout the entire process, from distillation to finished product, the flow of radioactivity is monitored with online silicon PIN diode detectors.

### Automatic Distillation

The first part of the process platform is used for the dry distillation of astatine. The ^211^At is embedded in the target material, which consists of remaining non-activated bismuth and aluminium. This solid compound, kept in a quartz glass container, is inserted into a quartz glass oven, pre-heated to *circa* 700 ^°^C by the tube furnace. After insertion of the target material, the quartz glass oven is promptly sealed with a flow-through glass stopper. The distillation is started as the tube furnace indirectly heats the target material via the quartz glass oven and container to a temperature above the boiling point of astatine (Bp 380 °C^15^). The volatized ^211^At escapes the target material, and the aluminium and bismuth of the target material remain as a solid/liquid mixture. The small amount of bismuth melts but does not boil (Mp 271 ^°^C, Bp 1560 °C^15^) while the bulk part of the aluminium remain solid (Mp 670 ^°^C, Bp 2467 °C^15^), which indicate that the temperature of the material does not reach the 700 ^°^C set on the tube furnace. The glass stopper in the oven is connected to a capillary that allows (dry) nitrogen gas to flow into the oven, and the front of the oven is connected to a three-way valve. During distillation, the valve is set to allow the volatilized astatine to pass through the valve, into a capillary that leads to a cryotrap (built into the synthesis module), which is cooled to between –40 and –50 °C with liquid nitrogen. The other end of the astatine capture capillary is connected to a reaction vial, which in turn is connected, through module-controlled valves, to several traps to capture any (potentially) over-distilled astatine. The traps are placed in front of the vacuum pump, which maintains low pressure in the system during the distillation process. During distillation, the nitrogen flow, the liquid nitrogen cooling, and the reduced pressure are all controlled with the software that controls the entire module.

The distillation process is typically complete within 25–35 s, reaching a reduced pressure of about –0.3 to –0.4 Bar. It is followed by system pressure equalization to atmospheric pressure under gentle nitrogen flow (20–50 ml/min), which takes 4–5 min. After this, the astatine is again solid, and it can be eluted from the cryotrap capillary with an organic eluent. At ambient pressure, the three-way valve on the quartz glass oven is adjusted to block the eluent from entering the oven. To elute the astatine, the capture capillary is washed with a solvent, such as chloroform or methanol in combination with an oxidant. The eluent is stored in one of the reagent storage containers and is transported through the astatine capture capillary (via module valves) with nitrogen flow, and it is captured in the reaction vial. At this point, the astatine is in a chemically useful form in the reaction vial. It can now either be used directly in the automated synthesis of a radiopharmaceutical in the synthesis module, or it can be removed for manual procedures in preclinical research.

**Figure 3:**
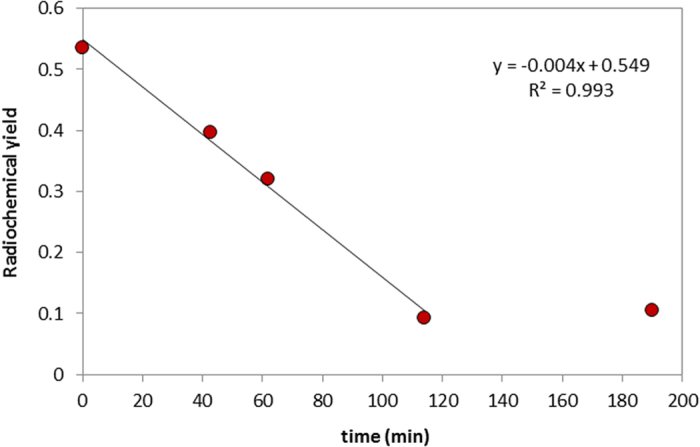
Time dependence of radiochemical yield (the amount of activity in the pure product compared to the amount employed in the reaction). The ATE-immunoconjugate (based on Trastuzumab) was labelled at different time intervals after At-211 was eluted with methanol, N-iodosuccinimde and acetic acid.

This distillation setup provided an average yield of 89 ± 2% (eluted activity compared to the activity of the target material) for the different eluents tested (Table 1). When eluting the activity with methanol combined with an oxidant for an organic diluent, it is of outmost importance to minimize the time interval between astatine elution and the start of synthesis to ensure a reasonably high yield of labelled compound (Fig. 3). This is most likely caused by a continuous transformation of astatine into a non-reactive specie, the nature of which will be the subject for further investigations in future work. After *circa* 120 minutes, the labelling yield in this case corresponds to unspecific binding of astatine to the antibody.

Astatine was produced in chloroform solution with the process platform and subsequently used successfully in numerous manual labelling experiments for both antibodies and peptides. As an example, labelling yield of prefabricated m-MeATE-immunoconjugates are 75% ± 5.5%, with 99% ± 0.8% radiochemical purity. This fact ensured that the astatine produced by the platform had adequate quality and usefulness. In those experiments, the chloroform was manually removed from the product by evaporation (method previously described^13^).

### Automatic Synthesis

Depending on the type of elution medium used, two different routes can be employed to continue synthesis after distillation. When the elution media is compatible with an aqueous matrix, like methanol or ethanol, e.g., for labelling conjugated antibodies or peptides, the reaction can start immediately. Minimizing the time after elution to the start of synthesis is, in this case, crucial for achieving a high yield of labelled product. When chloroform is used as the eluent, the astatine is stable in solution for several hours; this time window is practical for preclinical use, because it allows the activity to be apportioned among several different experiments. However, before continuing with direct synthesis in the synthesis module, the chloroform in the eluate must first be evaporated. Evaporation is performed on the platform by e.g. heating the astatine-chloroform solution in the reaction vial with a heating element, under a gentle nitrogen flow, for 5–10 min. This step results in less than 10% loss of radioactivity. Next, the dried astatine must be activated by adding a small amount of oxidant before radiolabelling can start. The up-scaling of this reaction has previously been investigated in detail^16^. All reagents required for the radio-synthesis can be stored in the reagent storage containers during the entire process (distillation and elution). These reagents are inserted into the reaction vial with the nitrogen gas flow. Nitrogen bubbling is also used to agitate the reaction solution. After the reaction, the product mixture is purified with size exclusion chromatography on an in-line column (liquid flows are facilitated with a syringe dispenser), followed by passing the product through a sterilization filter. The final, pure astatine-labelled product is collected in the product vial. The sterilization filter can be omitted for pre-clinical experiments.

During development, the reaction mixture was manually purified in parallel, to validate the efficiency of the in-line purification method (Table 2). When the process platform was used to label antibody conjugates with astatine, the yield after purification was 56 ± 5%, with a radiochemical purity of >95%. The antibodies, Trastuzumab and MX35, were conjugated to *N*-succinimidyl-3-(trimethylstannyl)benzoate (m-MeATE). These immunoconjugates had been prepared beforehand, according to the chelate method described above. The platform yield was lower than that achieved when prefabricated m-MeATE-immunoconjugates were manually labelled with astatine produced with the platform. Nevertheless, the platform result compared well with several other reported manual labelling methods^17^,^18^,^19^,^20^ and can produce immunoconjugates with high specific activity.

Astatine-labelled immunoconjugates produced with the process platform, after both chloroform and methanol elutions, were also tested in cell assays. Those studies showed that the process platform and the manual methods produced immunoconjugates with equivalent immunoreactivities. After chloroform elution, Trastuzumab-based immunoconjugates were tested on SKOV3 cells. The immunoreactive fractions (IRF) were 0.89 ± 0.16 for manual production and 0.91 ± 0.16 for automatic production. The MX35-based immunoconjugates were tested on OVCAR3 cells after methanol elution. Those products showed IRF = 0.78 ± 0.007 for manual production and IRF = 0.79 ± 0.03 for automatic production.

## Discussion

The PET techniques currently used widely for tumour imaging could not have been developed without the simultaneous development of automatic radiopharmaceutical production methods^19^,^20^. Therapy with short-lived radionuclides conjugated to different biomolecules is currently a new method that, similar to PET, requires automation before it can become a standard procedure in the clinic. Targeted alpha therapy with At-211 labelled antibodies has shown good results in early clinical trials; it is one of the more promising therapeutic methods available^6^,^7^. However, moving forward with this therapy requires automation. The new platform-based method described in this work enables automatic, reproducible, rapid, high-yield production of clinically relevant amounts of ^211^At and ^211^At -labelled radiopharmaceuticals. This process is fully automatic, from inserting the target material into the system to measuring the activity of the labelled product. A non decay-corrected yield of 50%, including At-211 distillation, elution, radiolabelling, and purification of the At-211- labelled antibody has been achieved. This automatic platform will improve and facilitate the implementation of radiopharmaceuticals based on the promising At-211 radionuclide.

Although the production of At-211 is currently limited, the precursor, Bi-209, is abundantly available; therefore, there is great potential for producing At-211 in large quantities for prospective clinical applications. However, an increase in the production of At-211 depends on both upgrading existing cyclotrons (facilities capable of producing this nuclide) and the construction of new cyclotron facilities.

An automated system is useful for standardizing radiopharmaceutical preparations and reducing the risk of human error. However, it will most likely be necessary to maintain trained radiochemists or radio-physicists to run the process. In its current state, the platform is a versatile research instrument, but it must be streamlined and adapted to radiopharmaceutical production according to GMP standards before it can be implemented and validated for clinical use. Although this transformation could be a complicated procedure regulatory wise the process platform in its current state already utilizes a GMP compliant software that would ease the process. The final product was in this work not tested for sterility or the presence of pyrogens, something that, however, also has to be included before using the product clinically. Astatine can be shipped within a 7–10 h transportation radius from cyclotron facilities. Thus, a large number of major hospitals within the radius of cyclotrons capable of producing clinical amounts of At-211 will be able to produce ^211^At-labelling radiopharmaceuticals for large scale clinical trials with the automated production platform presented here.

## Methods

### Process platform

**Figure 4:**
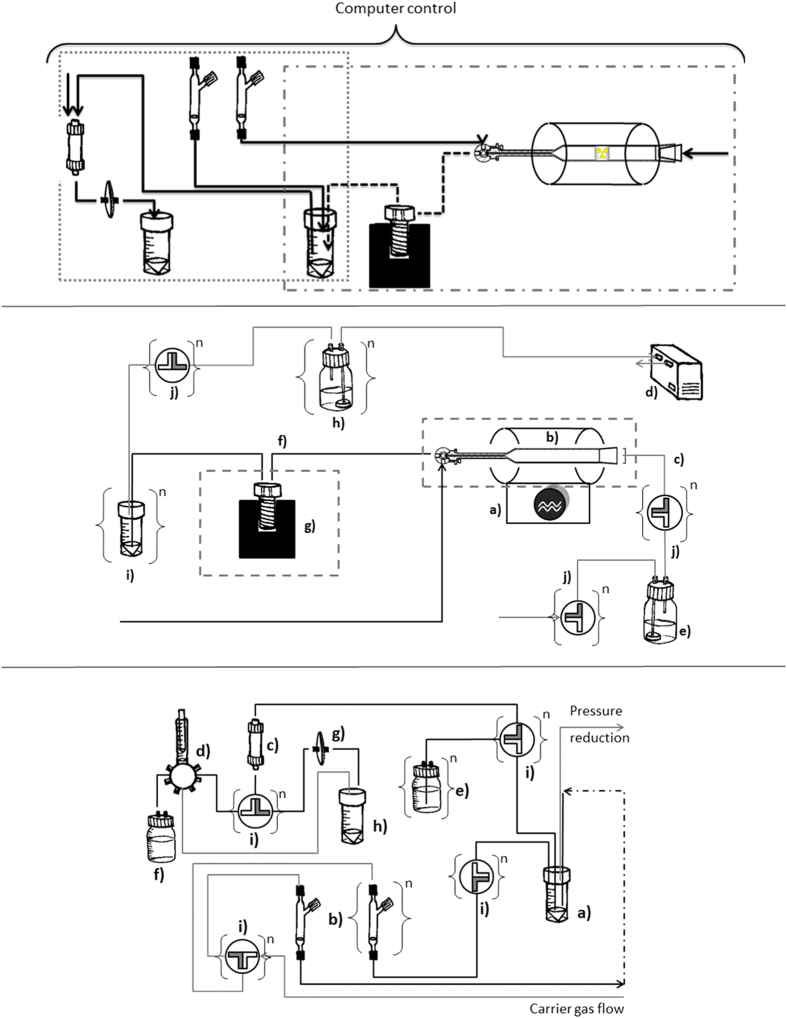
Detailed diagrams of process platform components. **Panel A (top)**: The process platform includes the distillation component (right box in dashed-dotted lines) and the synthesis/purification component (left, box in dotted lines) **Panel B (middle)**: The astatine distillation component of the platform includes: **a**) a tube furnace, **b**) a quartz glass oven **c**) carrier gas flow, **d**) a vacuum pump, **e**) a carrier gas dryer, **f**) an astatine condensation capillary, **g**) a cryotrap, **h**) a reaction vial, **i**) astatine capture traps, and **j**) three-way valves. The components outlined in dashed lines are shown in detail in Fig. 5. **Panel C (bottom)**: The astatine labelling/synthesis component of the automatic process platform includes: **a**) a reaction vial, **b**) sealed containers for reagent storage and introduction, **c**) a purification column, **d**) a syringe dispenser, **e**) containers for column buffers and reagents, **f**) a waste container, **g**) sterile filter option, **h**) a product vial, and **i**) three-way valves.

The process platform for the automated production of ^211^At-radiopharmaceuticals includes software-controlled equipment for astatine dry distillation (Fig. 4A, right) integrated with a synthesis module (Hot Box III, Scintomics Gmbh; Fig. 4A, left). Both components are controlled by the same software (Scintomics Control Centre).

#### Description of the Distillation component of the Process Platform

**Figure 5:**
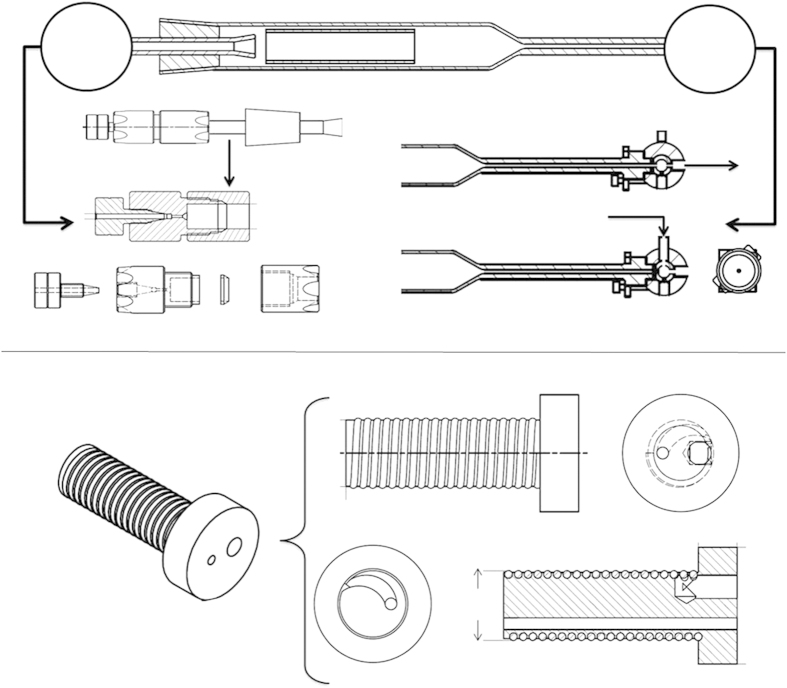
Detailed construction of key component parts. **Panel A (top)**: (*Top*) Dry distillation quartz glass oven with a tube that fits inside for inserting the target material. (*Lower left*) Details of how a PEEK capillary is connected to the quartz glass cone joint (OD = 8 mm). (*Lower right*) Three-way valve that can open and close passage from the oven outlet. **Panel B (bottom)**: (*Right*) Solid aluminium heat transfer insert for the cryotrap. (*Upper left*) The top part of the insert and (*Upper middle*) the capillary coiling along the outside. (*Lower left*) A cross-section of the insert and (*Lower middle*) the bottom part of the insert.

Figure 4B shows a detailed illustration of the integrated astatine distillation system for the automated process platform. The central part of the distillation system is a compact tube furnace (Carbolite® model MTF 10/25/130) that can produce heat >700 °C. The target consists of the backing (an aluminium slate of dimensions 30 × 27 × 5 mm) with a thin layer of bismuth (18 μm) and on top of this another thin layer of aluminium (7 μm) to prevent astatine evaporation during cyclotron production. The target material is removed from its backing by scraping off the activated top layer (<1 mm) using a hard-metal blade chisel and then transferred to a quartz glass container. The container is then inserted into a sealed, quartz glass oven (see Fig. 5A), which is heated by the tube furnace for astatine dry distillation. To facilitate efficient heat transfer from the tube furnace to the glass oven, the ratio between the oven outer diameter (OD) and the furnace tube inner diameter (ID) should be >0.8. The target material is inserted through an inlet joint of the quartz glass oven (left), which is connected to a capillary flow tube for the nitrogen carrier gas. The outlet of the quartz glass oven (right) is joined to a three-way valve, which is connected to capillary tubes for astatine condensation and for adding astatine transfer medium. The evaporated astatine is transferred from the quartz glass oven into the system with reduced pressure, created by a chemically resistant, software-controlled, vacuum pump (N810FT Laboport, KNF, capacity 10 l/min, ultimate vacuum <350 mbar) and the software-controlled nitrogen carrier gas flow (20–50 ml/min). The carrier gas is dried before entering the system with a scrubbing procedure that utilizes a moisture absorption media (iron-impregnated silica gel, Chameleon®). The evaporated astatine is condensed as a dry residue in a chemically inert, flexible capillary made of Fluorinated Ethylene Propylene (FEP) with OD 1/16” and ID 0.5–1 mm. The condensation is facilitated by directing the capillary into a software-controlled cryotrap (built into the synthesis module), with combined indirect cooling, with liquid nitrogen, and electrical heating. The cooling is best kept under –40 ^°^C. The cryotrap insertion for heat transfer to/from the astatine condensation capillary is illustrated in Fig. 5B. The back end of the astatine condensation capillary is joined to the reaction vial to collect the eluted astatine, and subsequently, the astatine is used for labelling. Several traps (volume expansion and sulphite gas scrubbing) are connected in series after the reaction vial to capture any potentially over-distilled gaseous astatine before entering the vacuum pump. The liquid and gas transport in the system are facilitated with the nitrogen carrier gas flow via 20, software-controlled, three-way valves in the synthesis module, unless otherwise stated.

The details of the ^211^At-dry distillation quartz glass assembly are illustrated in Fig. 5A. The outlet of the quartz glass distillation oven is joined to a three-way valve, which allows flow-through distillation and elution of At-211. On the distillation oven inlet, a modified quartz glass cone joint is attached to a capillary connection to allow carrier gas flow. The target material, which contains At-211, is inserted into a quartz glass tube, which is placed into the oven by removing the inlet cone joint. One setting of the three-way valve at the outlet allows evaporated astatine to pass from the oven into the condensation capillary. The other setting of the valve closes off the passage from the heated distillation glass, and opens between the reaction vial and the valve before the reagent storage container containing the solvent for safe elution of condensed astatine. The connection between the quartz glass cone joint at the inlet and the capillary for the carrier gas flow is adapted for a capillary with an OD of 1/8”, and it is constructed of a mechanically stable, heat-resistant, non-conducting plastic material, Polyether Ether Ketone (PEEK). The connection is also made gas tight with Teflon packing.

Figure 5B shows the solid heat transfer insert for the cryotrap. It is made out of heat-conducting aluminium, and it is adapted to hold a 1/16” chemically-inert, flexible FEP plastic capillary. The cooling of the insert and capillary assembly enables the condensation of astatine, which forms a dry residue. The ratio between the heat transfer insert OD and the cryotrap well ID should be preferably >0.9 to allow for efficient indirect cooling/heating.

The distillation setup described in Figs 4 and 5 enabled fast, reproducible, remote-controlled astatine distillation from the target material, with short heating times (<1 min), followed by approximately 5 min for equalizing the system pressure.

#### Production of Astatine in a Chemically Useful Form with the Process Platform

The process platform described can be used for software-controlled astatine dry distillation. It delivered astatine in a chemically useful form for further processing. This form can be used in automated labelling procedures or for manual procedures.

Target material that typically contained between 550 and 650 MBq ^211^At, produced from alpha particle irradiation (28 MeV) of ^209^Bi (^211^At (α,2n) ^209^Bi) was removed from the target backing. The quartz glass oven was heated to circa 700 °C, with the three-way valve in the flow-through position, and a gentle nitrogen flow (20 ml/min) through the system. Simultaneously, with pre-heating of the distillation glassware the cryotrap was cooled to *circa* −50 °C with liquid nitrogen. The automatic distillation was started directly after the target material was inserted into the distillation glassware. The software started the vacuum pump, which reduced the pressure in the system, as the nitrogen flow was increased to 50 ml/min. Short distillation times were used (<1 min), and when the vacuum pump was turned off, the pressure was –0.3 to –0.4 Bar. Nitrogen flow was maintained to slowly increase the pressure in the system to atmospheric pressure. Upon atmospheric pressure, the three-way valve was set to the elution position, and the astatine transfer liquid (V_tot_ = 120 μl) was introduced to elute the astatine, under a gentle nitrogen flow (5–10 ml/min), from the condensation capillary into in the reaction vial. The transfer liquids were chloroform (CHCl_3_) and a methanol solution with 0.4% acetic acid and 8 μg/ml N-Iodo succinimide (MeOH/NIS; Table 1).

#### Description of the Synthesis component of the Process Platform

Figure 4C shows the astatine labelling component of the process platform. In the reaction vial, astatine was collected after elution from the condensation capillary. Elution was performed with an organic astatine transfer medium, such as chloroform or methanol, with added N-iodo-succinimide. This medium was stored in a sealed container during distillation. The transfer medium was introduced into the three-way valve on the outlet of the distillation-oven, and it entered the condensation capillary through a chemically-inert capillary with the software-controlled nitrogen gas flow (flow rate between 15–10 ml/min). It is particularly important to use chemically inert capillaries when using chloroform in the elution medium, because chloroform can degrade the tubing, which allows unwanted chemicals to elute into the synthesis vial. Several other capillaries were connected to the reaction vial for:

1- creating reduced pressure in the system with the vacuum pump,

2- introducing labelling reagents with the nitrogen gas flow, and

3- transporting product to the purification column with the software-controlled syringe dispenser (ceramic, 9 gates).

The labelling reagents were stored in sealed containers on the module (V_tot_ = 1–5 ml). However, for small liquid volumes, down to 0.1 ml, which have high surface tension (i.e., water based), small pieces of 1/4” FEP tubing (<4 cm) were used as containers to ensure the liquid loss was below 15 volume %. The in-line purification column was a HighTrap or a PD10 gel filtration column (Sephadex G25, GE Healthcare) for protein purification. In addition, several purification buffers and reagents were added to the column with the syringe dispenser on the module. During equilibration of the column, effluent liquids were directed to a waste container. The sterilization filter placed between the column and the final product vial can be omitted, when the product does not require clinical grade purity.

#### Production of Astatine Labelled Antibodies with the Process Platform

The process platform was used to synthesize astatinated antibodies. This was achieved by labelling prefabricated ε-Lysyl-3-(trimethylstannyl)benzamide (MeATE)-trastuzumab antibody conjugates in an astatodestannylation reaction. The MeATE-conjugates were prepared as described previously^13^. The astatine used in these experiments was produced in the automatic distillation part of the process platform (Fig. 4A). Depending on the astatine transfer medium used, the synthesis was performed with different methods.

Method 1: When the astatine transfer medium (V_tot_ = 120 μl) consisted of a methanol solution with 0.4% acetic acid and 8 μg/ml N-Iodo succinimide (NIS) as oxidant, the astatine was eluted directly into a solution of the conjugated target antibody to start the labelling reaction. In this case the ATE-modified antibody Trastuzumab (1 mg/ml, V_tot_ = 520 μl) was automatically introduced into the reaction vial prior to astatine elution. After a reaction time of 1 min, where agitation was facilitated with nitrogen gas bubbling, residual tin groups were removed with an excess of NIS in citrate buffer (pH 5.5, with 3% methanol and 1% acetic acid, V_tot_ = 110 μl). After an additional 1 min, the reaction was quenched with sodium ascorbate (6 mg/ml, V_tot_ = 110 μl).

Method 2: When the astatine was eluted from the cryotrap with chloroform, the solvent had to be evaporated before starting the reaction. This was achieved on the automatic process platform by reducing the pressure or by heating under a nitrogen gas flow. The evaporation step resulted in activity losses <10%. In this case, the oxidizing agent, NIS (8 μg/ml in methanol with 0.4% acetic acid, V_tot_ = 120 μl), had to be added to the dry astatine residue in the reaction vial, prior to introducing the immunoconjugate (ATE-modified antibody Trastuzumab, 1 mg/ml, V_tot_ = 520 μl). The next reactions follow the description in Method 1.

The following steps were the same in both methods. Reagent introductions were facilitated with a gentle nitrogen flow (5–15 ml/min) from sealed reagent containers via 4 three-way valves. The product was purified either automatically, by setting up a HiTrap or PD10 Desalting (Sephadex G25, GE Healthcare) column in-line, or manually, with a NAP10 column (Sephadex G25, GE Healthcare).

### Quality control

The quality of the products synthesized with the process platform was investigated in two different ways. In both cases, activity was counted with a NaI(Tl) γ-counter (Wizard 1480, Wallac, Finland).

#### Radiochemical Purity

The radiochemical purity (RCP) of the astatine labelled immunoconjugates was determined with methanol precipitation. Labelled product (1–15 kBq) was added to 200 μl of PBS, with 1% bovine serum albumin (as the carrier protein) and 0.05% sodium azide. Each sample was prepared in triplicate. Then, 500 μl of methanol was added to precipitate the proteins, and the total activity in each sample was measured. The samples where then centrifuged (4400 rpm, 15 min), the supernatants were withdrawn, and the activities in the remaining pellets were measured. The RCP was calculated as the fraction of protein-bound radioactivity compared to the total amount applied.

#### Antibodies and cell lines

Immunoconjugates were produced with the monoclonal antibodies, Trastuzumab and MX35. Trastuzumab is specific for ErbB2 (Her2); it was obtained from the Swedish Pharmacy (Sahlgrenska University Hospital, Göteborg, Sweden). MX35 is a murine IgG1 monoclonal antibody, specifically directed toward a membrane phosphate transporter protein (NaPi2b), which is expressed in over 90% of human epithelial ovarian cancers^21^. The hybridoma cells for MX35 were provided by the Ludwig Institute (New York, NY, USA), and they were cultured at the Department of Cell and Molecular Biology at the University of Gothenburg (Sweden). The human tumour cell lines, SKOV3 (expressing Her2) and NH:OVCAR3 (expressing NaOi2B) were used for the cell assays. Both cell lines were obtained from ATTC (Rockville, MD, USA).

#### Immunoreactive Fractions

The immunoreactive fractions were determined according to a well-establ-ished method previously described^24^. In short, a single-cell suspension of SKOV3 cells (5 × 10^6^ cells/mL) was serially diluted (1:2) six times. To these cell samples (duplicate samples), a constant amount of ^211^At-labelled Trastuzumab (5–10 ng) was added, and the mixture was allowed to react for 180 min with gentle agitation in room temperature. Cells were then centrifuged, and the pellets were washed with PBS. The bound fraction was determined by measuring the activity associated with the cells (B), compared to the total radioactivity (T) added to each sample, determined through reference samples. The immunoreactive fraction (IRF) B_max_ was determined by nonlinear regression analysis (least squares method) of the average data points (B/T) for each serial sample from the binding assay.

## Additional Information

**How to cite this article**: Aneheim, E. *et al.* Automated astatination of biomolecules — a stepping stone towards multicenter clinical trials. *Sci. Rep.* **5**, 12025; doi: 10.1038/srep12025 (2015).

## Figures and Tables

**Table 1:** Examples of the yields of dry distilled At-211 (in a chemically useful form) achieved, when eluted with different media on the automatic process platform.

Target material activity (MBq)	Elution media	Distillation Yield (%)
626	MeOH/NIS	87
594	CHCl_3_	85
572	MeOH/NIS	88
593	CHCl_3_	90
442	MeOH/NIS	92
623	CHCl_3_	91

Activity quantification made using a CRC Capintec 15 R dose calibrator.

**Table 2:** Yields and purity of At-211-labelled antibodies prepared with the automated process platform and purified either manually or on the platform; the antibodies were previously prepared as m-MeATE-conjugates.

Initial activity (MBq)	Elution medium	Conjugated Antibody	Purification Method	Labelling yield (%)	Radiochemical Purity (%)	Specific activity (MBq/mg)
509	MeOH/NIS	Trastuzumab	Manual Nap10	55	97	560
456	MeOH/NIS	Trastuzumab	Automatic HiTrap	48	99	430
400	MeOH/NIS	MX35	Automatic PD10	56	95	446
219	CHCl_3_*	Trastuzumab	Manual Nap10	64	96	280
215	CHCl_3_*	Trastuzumab	Automatic HiTrap	55	98	230

^*^CHCl_3_ was evaporated on the process platform before starting synthesis.
